# EFFECTS OF REMOVING VISUAL INPUTS ON POSTURAL SWAY COMPLEXITY IN YOUNG ADULTS AND OLDER ADULTS WITH AND WITHOUT T2D

**DOI:** 10.1093/geroni/igac059.2913

**Published:** 2022-12-20

**Authors:** Kellyn Jason, Junhong Zhou, Wanting Yu, Brad Manor, Azizah Jor’dan

**Affiliations:** University of Massachusetts Boston, Boston, Massachusetts, United States; Harvard Medical School/Hebrew SeniorLife, Roslindale, Massachusetts, United States; Hebrew SeniorLife, Roslindale, Massachusetts, United States; Hebrew SeniorLife/Harvard Medical School, Roslindale, Massachusetts, United States; University of Massachusetts, Boston, Massachusetts, United States

## Abstract

Standing postural control is complex and involves sensory inputs from the visual, vestibular, and somatosensory systems. Postural sway complexity, as often assessed by multiscale entropy, has been linked to the ability of the postural control system to adapt to “stressors,” including the removal of visual inputs. We aimed to determine the effects of removing visual inputs (i.e., eyes closed, EC) on postural sway complexity in young adults (YA), and older adults (OA) with and without type 2 diabetes mellitus (T2DM). Twenty-five YA (age=21–30 years), 25 OA (age=70–90 years), and 19 T2DM (age=67–93 years) stood for 30 seconds under two conditions – eyes open (EO), and EC for three trials. Postural sway acceleration was measured in the medial-lateral (ML) and anterior-posterior (AP) direction during EO and EC conditions. Sway complexity was quantified using multi-scale entropy. The YA group had a significant reduction in ML and AP sway complexity during EC when compared to EO (p=0.04 and p < 0.001, respectively). Though not significant, the OA group exhibited a trend towards increased ML sway complexity during EC compared to EO standing (p=0.07). No significant changes in AP sway complexity were observed in OA or T2DM, or in ML sway complexity in T2DM. The dynamics of postural control differ between YA, OA, and T2DM groups. There was a significant difference in postural sway complexity between the EO and EC among YA, with no differences in OA and T2DM between conditions. The lack of change suggests maladaptation to stressors, such as vision loss.

